# Impact of Clinical Decision Support with Mandatory versus Voluntary Venous Thromboembolism Risk Assessment in Hospitalized Patients

**DOI:** 10.1055/s-0044-1790519

**Published:** 2024-09-12

**Authors:** Vinita Bahl, Marc J. Moote, Hsou Mei Hu, Darrell A. Campbell

**Affiliations:** 1 Department of Surgery, University of Michigan Health Michigan Medicine, Ann Arbor, Michigan, United States; 2 Office of Clinical Affairs, University of Michigan Health Michigan Medicine, Ann Arbor, Michigan, United States; 3 Section of Plastic Surgery, Department of Surgery, University of Michigan Health Michigan Medicine, Ann Arbor, Michigan, United States; 4 Section of Transplant Surgery, Department of Surgery, University of Michigan Health Michigan Medicine, Ann Arbor, Michigan, United States

**Keywords:** venous thromboembolism, risk factors, prophylaxis, clinical trials, deep vein thrombosis, pulmonary embolism

## Abstract

**Background** Venous thromboembolism (VTE) causes significant preventable morbidity and mortality in hospitalized patients. Assessing VTE risk is essential to initiating appropriate prophylaxis and reducing VTE outcomes. Studies show that computerized clinical decision support (CDS) can improve VTE risk assessment (RA), prophylaxis, and outcomes but few examined the effectiveness of specific design features.

From 2008 to 2016, University of Michigan Health implemented CDS for VTE prevention in four stages, which alternated between voluntary and mandatory RA using the 2005 Caprini model and generated inpatient orders for risk-appropriate prophylaxis based on CHEST guidelines. This cross-sectional study evaluated the impact of mandatory versus voluntary RA on VTE prophylaxis and outcomes for adult medical and surgical patients admitted to the health system.

**Methods** Interrupted time series analysis was conducted to evaluate the trend in smart order set-recommended VTE prophylaxis by CDS stage. Logistic regression with CDS stage as the primary independent variable was used in pairwise comparisons of VTE during hospitalization and within 90 days post-discharge for mandatory versus voluntary RA. Adjusted odd ratios (ORs) were calculated for total, in-hospital, and post-discharge VTE.

**Results** In this study of 223,405 inpatients over 8 years, smart order set-recommended prophylaxis increased from 65 to 79%; it increased significantly when voluntary RA in Stage 1 became mandatory in Stage 2 (10.59%, *p* < 0.001) and decreased significantly when it returned to voluntary in Stage 3 (−11.24%, *p* < 0.001). The rate increased slightly when mandatory RA was reestablished in Stage 4 (0.23%, *p* = 0.935).

Adjusted ORs for VTE were lower for mandatory RA versus adjacent stages with voluntary RA. The adjusted OR for Stage 2 versus Stage 1 was 14% lower ( *p* < 0.05) and versus Stage 3 was 11% lower ( *p* < 0.05). The adjusted OR for Stage 4 versus Stage 3 was 4% lower ( *p* = 0.60).

These results were driven by changes in in-hospital VTE. By contrast, the incidence of post-discharge VTE increased in each successive stage.

**Conclusion** Mandatory RA was more effective in improving smart order set-recommended prophylaxis and VTE outcomes, particularly in-hospital VTE. Post-discharge VTE increased despite high adherence to risk-appropriate prophylaxis, indicating that guidelines for extended, post-discharge prophylaxis are needed to further reduce VTE for hospitalized patients.

## Introduction

Venous thromboembolism (VTE), which includes deep vein thrombosis (DVT) and pulmonary embolism (PE), is a common complication and leading cause of morbidity and mortality among hospitalized patients. Approximately 10% of hospital deaths are attributed to PE, and patients who survive have an increased risk of postthrombotic syndrome, pulmonary hypertension, or recurrent thrombosis. 1

Hospital-associated VTE (HA-VTE) is commonly defined as VTE that occurs during hospitalization or within 90 days after hospital discharge. The majority of HA-VTE events occur after patients are discharged. 2 3 Randomized clinical trials established that prophylactic medications and mechanical devices can significantly reduce DVT and PE in hospitalized surgical and medical patients. Despite this evidence, numerous studies show that prophylaxis is underutilized or not effectively targeted to patients at greatest risk. 4 5

Since VTE causes significant preventable morbidity and mortality, federal health care organizations including the Centers for Medicare & Medicaid Services, the Centers for Disease Control and Prevention, and the Agency for Healthcare Research and Quality made VTE prevention a priority. 6 7 8 In 2008, the Surgeon General issued a call to action to prevent DVT and PE and in 2020 the American Heart Association (AHA) renewed the call specifically for hospitalized patients. 9 10

Accurate assessment of patient VTE risk is critical to improving prophylaxis among high-risk patients and reducing unnecessary prophylaxis in low-risk patients. VTE guidelines are available to help clinicians stratify patients into low-, moderate-, and high-risk levels and prescribe risk-appropriate prophylaxis but are used inconsistently or improperly. 11 Computerized clinical decision support (CDS) tools are a means to operationalize guidelines and have been shown to improve VTE prophylaxis rates and reduce VTE outcomes among hospitalized patients. 12 13 14 15 16 CDS tools, defined as a point-of-care electronic tool to help clinicians assess patient-specific VTE risks and prescribe risk-appropriate prophylaxis, are varied in their design as well as short- and long-term impact. 17

Some CDS tools automatically assess patient VTE risk, based on risk factors documented in the electronic medical record, and then send alerts to clinicians identifying high-risk patients and recommending appropriate prophylaxis. 18 With this type of CDS, rates of appropriate prophylaxis improve initially but tend to decline over time owing to alert fatigue.

Other CDS tools require clinicians to select VTE risk factors from a checklist to calculate the risk for each patient. This method was viewed as a means to counteract alert fatigue but was also found to underestimate risk scores. 19 20

A meta-analysis identified several features associated with the most effective CDS in surgical populations, including integration into the provider workflow, mandatory requirement to conduct patient VTE risk assessments, prepopulating orders for guideline-recommended prophylaxis, but allowing the provider to change the order (i.e., opt-out provision). 21 However, few studies have directly tested the effectiveness of these CDS design features in improving VTE prophylaxis and reducing adverse outcomes.

Prior to implementing CDS for VTE prevention, University of Michigan Health (UMH) adopted the Caprini VTE risk assessment model (RAM) and providers scored patient risk using a preprinted worksheet. Although the worksheet was used infrequently, patients with a formal risk assessment were more likely to receive VTE prophylaxis, a finding reported by several published studies. 22 23

In 2008, UMH implemented CDS by adding the worksheet to admission order sets within a computerized order entry system (CPOE). After providers checked applicable VTE risk factors, the CDS computed the Caprini risk score, risk level, and presented a recommendation for prophylaxis based on CHEST guidelines. 1 24

In this initial CDS, VTE risk assessment was voluntary. About a year later, it was mandated via a “hard stop” and coupled with an opt-out feature to compel providers to select the smart order set-recommended prophylaxis. Five years later, a new electronic health record (EHR) was installed and CDS VTE risk assessment was replaced with a voluntary system because of technical limitations of the new EHR. About a year later, the hard stop was reestablished.

UMH employed a multifaceted approach to VTE prevention, combining CDS with quality improvement (QI) programs (see the Supplementary Material Tables S1 and S2 for details about the features of the UMH CDS and of QI programs).

Using data from UMH, we sought to measure and analyze the impact of implementation and subsequent alternation of voluntary and mandatory VTE risk assessment on rates of VTE prophylaxis and outcomes for hospitalized surgical and medical patients. Our results shed light not only on the effect of mandatory risk assessment but also on the challenges of reducing the burden of HA-VTE.

## Methods

### Study Design

A cross-sectional analysis was conducted using clinical data from UMH. This study examines the impact of the changes in CDS on rates of VTE prophylaxis and outcomes over 8 years from May 2008 through July 2016, covering four distinct stages of CDS implementation:

Stage 1: voluntary risk assessment embedded in an Eclipsys CPOE (14 months, beginning May 2008).
Stage 2: mandatory risk assessment in the same CPOE (4 years 10 months, beginning August 2009).
Stage 3: voluntary risk assessment in an Epic EHR (14 months, beginning June 2014).
Stage 4: mandatory risk assessment in the same EHR (12 months, beginning August 2015).

Throughout the 8-year study period, the CDS for VTE prevention was based on the Caprini RAM. Although first developed for surgical populations, we applied the Caprini RAM to both medical and surgical patients, since: (1) the Caprini RAM was one of only a few models available at the time we first introduced a formal program to improve VTE risk assessment, (2) the Caprini RAM is comprehensive and includes risk factors that are important predictors of VTE that are not in other RAM, like family history of VTE and obstetrical complications, and (3) as a practical matter, it was important to establish consistent CDS implementation using one tool as we sought to mandate VTE risk assessment and appropriate prophylaxis for all adult inpatients.

The effect of each of the four stages of CDS was assessed by analyzing rates of:

- Documented VTE risk assessment, measured as the percentage of patients for whom the checklist of risk factors was used to calculate and record a patient's risk score and risk level.
- VTE prophylaxis, both risk-appropriate and insufficient (under- or delayed) prophylaxis. Risk-appropriate VTE prophylaxis is measured as the percentage of patients with orders consistent with UMH smart order set-recommended prophylaxis based on the documented risk level. Detailed definitions are provided in Table 1 .

**Table 1:** Definition of UMH smart order set-recommended and insufficient VTE prophylaxis

Risk level (risk score)	Smart order set-recommended VTE prophylaxis a b	Insufficient VTE prophylaxis
Low (0 or 1)	None	N/A
Moderate (2)	• SQ UF heparin TID or • SCD if contraindication to chemoprophylaxis c	• SQ UF heparin BID or • Smart order set-recommended prophylaxis ordered, but beyond time criteria d • No SCD with contraindication to chemoprophylaxis (i.e., no prophylaxis)
High (3 or 4)	• SQ UF heparin TID with or without SCD or • LMWH in prophylactic doses (30 or 40 mg daily or 30 mg BID) with or without SCD OR • SCD if contraindication to chemoprophylaxis c	• SQ UF heparin BID or • Smart order set-recommended prophylaxis ordered, but beyond time criteria d • No SCD with contraindication to chemoprophylaxis (i.e., no prophylaxis)
Highest (5 + )	• SQ UF heparin TID or • LMWH in prophylactic doses (30 or 40 mg daily or 30 mg BID) AND • SCD if surgical patient (optional for medical patients) OR • SCD if contraindication to chemoprophylaxis c	• SQ UF heparin BID, or • No SCD in surgical patient, or • Smart order set-recommended prophylaxis ordered, but beyond time criteria d • No SCD with contraindication to chemoprophylaxis (i.e., no prophylaxis)

Abbreviations: BID, two times per day; LMWH, low-molecular-weight heparin; mg, milligrams; SCD, sequential compression device; SQ UF, subcutaneous unfractionated; TID, three times per day.

a Medications for orthopedic surgery are different from those listed and are specific for orthopedic surgery. Providers wrote orders for these medications using the CDS opt-out function until a year after the study period ended, when a specialty-specific order set was implemented.

b The duration of prophylaxis was based on 2008 and 2012 CHEST guidelines. 1 23

c Contraindication documented in clinical decision support system.

d Time criteria: prophylaxis ordered within 2 days following admission or operation.

During the study, we rigorously followed 2008 and 2012 CHEST guidelines to establish UMH smart order set recommendations for the type of VTE prophylaxis by risk level and the duration of prophylaxis, which advised inpatient prophylaxis to be discontinued at discharge for all except a few populations where extended prophylaxis was deemed necessary (e.g., hip fracture and total hip or knee arthroplasty). 1 24

Studies of the Caprini risk score and outcomes in several patient subgroups demonstrated that a more favorable benefit/harm ratio for chemoprophylaxis can be achieved with higher Caprini risk score thresholds for chemoprophylaxis that are specific for each subgroup (e.g., general surgery patients, acutely ill medical patients). 25 26 27 Although a few were published during our study period, their findings were not reflected in the CHEST guidelines. We therefore did not change our risk score threshold for smart order set-recommended VTE chemoprophylaxis for different patient subgroups.

- VTE outcomes, defined as clinically diagnosed, image-confirmed, lower extremity DVT and PE during hospitalization or within 90 days after hospital discharge. VTE outcomes were identified from positive test results in radiology imaging reports using an internally validated computerized text search method; VTE incidence was reported in total and as in-hospital or post-discharge VTE. Upper extremity DVTs were not counted because the pathophysiology and strategies for preventing upper extremity versus lower extremity DVT are different. 28 VTE events that occurred during the index hospitalization must have occurred on the third day of the hospital stay or later to be included.

### Study Population

Patients included in the study were adults admitted to UMH from May 2008 through July 2016. Patients excluded from the study were those meeting any of the following criteria: (1) age younger than 18 years, (2) admitted to ophthalmology, obstetrics, psychiatry, or rehabilitation services, (3) admitted under observation status, (4) received therapeutic doses of anticoagulants for any reason upon admission, and (5) had a diagnosis of VTE upon admission.

### Statistical Analysis

Statistical analyses were conducted to evaluate the rate of documented risk assessment by stage and differences in rates of prophylaxis between patients who were and were not risk-assessed. In addition, because CDSs that employ checklists to calculate patient VTE risk have been found to underestimate risk scores, the reliability of documented patient risk levels was tested. For each patient, the documented risk level was compared to the risk level calculated from data about risk factors in hospital information systems.

Interrupted time series analyses were conducted to evaluate the trends in smart order set-recommended and in insufficient VTE prophylaxis by CDS stage. Results were reported as changes in the level and trend of rates of prophylaxis following implementation of each CDS stage. 29 The model was tested for autocorrelation using the Durbin–Watson statistics.

Logistic regression was used to estimate the odds of VTE during hospitalization or within 90 days after discharge by CDS stage for total, in-hospital, and post-discharge VTE. Pairwise comparison of the odds ratio of VTE during hospitalization or within 90 days after discharge for CDS stages with mandatory versus voluntary risk assessment for total, in-hospital, and post-discharge VTE was conducted based on the Wald test.

In the models, the CDS stage was the primary independent variable. Four dichotomous control variables were included. Length of hospitalization of 3 days or longer was added to control for unobserved factors associated with short stays on VTE outcomes. A variable for surgery was included to control for differences in the pathogenesis of VTE in surgical versus medical patients. Transfers to UMH from another acute care hospital and discharges to another facility were included to control for unobserved pre-admission and post-discharge care in other facilities on VTE outcomes. Both models were tested using the Hosmer–Lemeshow goodness-of-fit test. *p-* Values were two-tailed, and significance was set at *p* <0.05. Analysis was conducted using SAS version 9.4 (SAS Institute, Cary, North Carolina, United States) and Stata version 14.1 (StataCorp, College Station, Texas, United States).

Finally, we examined differences in adjusted VTE incidence rates per 10,000 by CDS stage for patients with smart order set-recommended prophylaxis and patients with orders for insufficient prophylaxis.

During the initial data analysis, we observed an increase in incidence of post-discharge VTE from one stage to the next. Consequently, we examined incidence of post-discharge VTE in two study population subgroups, surgical and medical, and evaluated potential contributing factors, including trends in lengths of stay, risk scores, and prevalence of individual risk factors.

This study was written following the Strengthening the Reporting of Observational Studies in Epidemiology (STROBE) checklist for research reporting of observational studies. 30 The study was approved by the University of Michigan Medical School Institutional Review Board.

## Results

Results from the analysis of a total study population of 223,405 adult inpatients over a period of 8 years demonstrate that mandatory risk assessment is more effective than voluntary risk assessment in improving adherence to smart order set-recommended prophylaxis and reducing rates of VTE, particularly in-hospital VTE. The total study population consisted of 62% medical and 38% surgical patients (see the Supplementary Material Table S3 for a description of the characteristics of the study population).

The percentage of patients with a documented risk assessment grew from Stage 1 through Stage 4. After initial CDS implementation in Stage 1, it was 42.4% but then grew substantially and in Stage 4 it was almost 98%. Regardless of stage of CDS implementation, patients with a documented risk assessment were significantly more likely to receive an order for VTE prophylaxis than patients without ( Table 2 ).

**Table 2:** Patients with documented VTE risk assessment and impact on prophylaxis rates

		Percent any VTE prophylaxis		
CDS stage ( *n* )	Percent patients w RA	Patients w RA	Patients wo RA	*p* -Value
Stage 1 ( *n* = 29,520)	42.4%	96.5%	81.5%	<0.001
Stage 2 ( *n* = 134,013)	91.5%	98.7%	75.1%	<0.001
Stage 3 ( *n* = 32,239)	91.5%	98.0%	78.7%	<0.001
Stage 4 ( *n* = 27,633)	97.7%	98.9%	72.1%	<0.001

Abbreviations: CDS, clinical decision support; *n* , number; RA, risk assessment; VTE, venous thromboembolism; w, with; wo, without.

We evaluated the documented risk levels by stage and found that they matched the risk level calculated from hospital information systems in 46 to 56% of patients. The level of agreement between the two risk scoring methods is not very strong, with weighted kappa scores between 0.436 and 0.482. When the documented risk level did not match, it was most often found to be lower than the score from hospital information systems.

The analysis of the impact of each CDS stage on adherence to smart order set-recommended prophylaxis using segmented regression of time series data identified several stages when significant changes occurred.

**Fig. 1:**
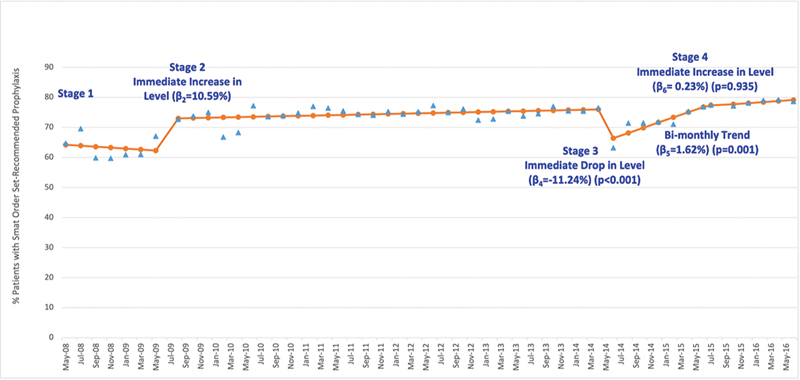
Percentage of study population with smart order set-recommended prophylaxis. Change in percentage of study population with orders for smart order set-recommended prophylaxis ( *y* -axis) versus calendar quarter of the study period ( *x* -axis) shown for actual percentage (  ) and predicted percentage (  ). R-square = 0.803, Durbin–Watson statistics = 1.920 (close to 2—insignificant autocorrelation)

The introduction of Stage 2 and the hard stop significantly increased the level of smart order set-recommended prophylaxis. The transition to Stage 3, which removed the hard stop, produced a significant decline in the percentage of patients with orders for recommended prophylaxis. After Stage 3, the percentage exhibited a significant upward trend and, when the hard stop was reintroduced in Stage 4, the increase in level was not significant. During the study period, adherence to smart order set-recommended prophylaxis grew from 65 to 79% ( Fig. 1 ).

**Fig. 2:**
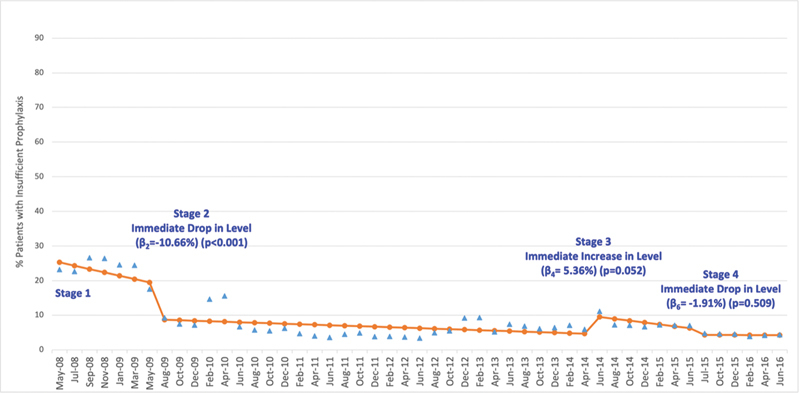
Percentage of study population with orders for insufficient prophylaxis. Change in percentage of study population with orders for insufficient prophylaxis ( *y* -axis) versus calendar quarter of the study period ( *x* -axis) shown for actual percentage (  ) and predicted percentage (  ). R-square = 0.902, Durbin–Watson statistics = 1.793 (close to 2—insignificant autocorrelation)

Changes in the percentage of patients with orders for smart order set-recommended prophylaxis when Stages 2 and 3 were implemented were accompanied by opposite changes in the percentage of patients with orders for insufficient prophylaxis. That is, the level of insufficient prophylaxis fell with the introduction of hard stop requiring risk assessment (Stage 2) and increased with Stage 3, when the hard stop was removed. The increase at Stage 3, however, did not achieve statistical significance ( Fig. 2 ).

Our analysis of the impact of each CDS stage on VTE outcomes showed that adjusted rates of VTE were higher for Stages 1 and 3, where risk assessment was voluntary compared to stages where it was mandatory. Adjusted rates of in-hospital VTE followed a similar pattern, but adjusted rates of post-discharge VTE rose from one stage to the next ( Table 3 ). Post-discharge VTE accounted for about half of VTE in Stage 1 and grew to more than 70% of all VTE in Stage 4.

**Table 3:** Adjusted VTE rates by CDS stage

	Adjusted VTE rate (95% CI)		
CDS stage	Total VTE	In-hospital VTE	Post-discharge VTE
Stage 1	1.11% (1.00–1.23%)	0.55% (0.47–0.64%)	0.56% (0.48–0.65%)
Stage 2	0.92% (0.87–0.97%)	0.33% (0.33–0.36%)	0.59% (0.55–0.63%)
Stage 3	1.13% (1.01–1.24%)	0.41% (0.34–0.48%)	0.72% (0.63–0.82%)
Stage 4	1.07% (0.94–1.19%)	0.27% (0.21–0.33%)	0.80% (0.69–0.90%)

Abbreviations: CDS, clinical decision support; CI, confidence interval; VTE, venous thromboembolism.

Similarly, results from the logistic regression pairwise comparison of VTE outcomes between successive CDS stages showed that the adjusted odds ratio for VTE was lower for stages with mandatory compared to voluntary risk assessment ( Table 4 ).

**Table 4:** Pairwise comparison of odds of VTE between stages of CDS implementation

	Adjusted odds ratio (95% CI)		
CDS Stage	Total VTE ^a^	In-hospital VTE ^b^	Post-discharge VTE ^c^
Stage 1 vs. Stage 2	0.859 d (0.760–0.971)	0.639 e (0.533–0.766)	1.070 (0.904–1.266)
Stage 3 vs. Stage 2	0.888 d (0.789–0.999)	0.930 (0.764–1.132)	0.848 d (0.732–0.982)
Stage 3 vs. Stage 4	0.959 (0.821–1.121)	0.712 d (0.534–0.950)	1.101 (0.914–1.327)
Stage 1 vs. Stage 4	0.928 (0.791–1.089)	0.489 e (0.371–0.647)	1.390 d (1.134–1.704)

Abbreviations: CDS, clinical decision support; CI, confidence interval; VTE, venous thromboembolism.

Note: Hosmer–Lemeshow goodness-of-fit test ( ^a^ *p* = 0.397; ^b^ *p* = 0.650; ^c^ *p* = 0.682).

d *p* < 0.05.

e *p* < 0.001.

The adjusted odds ratio for VTE was 14% lower for mandatory risk assessment in Stage 2 compared to voluntary risk assessment in Stage 1 and 11% lower for Stage 2 compared to voluntary risk assessment in Stage 3. These differences were statistically significant. The difference in odds ratio for mandatory risk assessment in Stage 4 compared to mandatory risk assessment in Stage 3 was not statistically significant. Finally, when we assess change spanning the study period by comparing the first to the last stage, the odds ratio for VTE was 7% lower in Stage 4 compared to Stage 1 due to significantly lower odds ratio of in-hospital VTE and despite a significantly higher odds ratio of post-discharge VTE.

The increase in incidence of post-discharge VTE for the total study population was driven by the medical patient subgroup ( Table 5 ). Within the medical subgroup, hospital lengths of stay increased from stage 1 to 4 (from 5.3 to 6.4 days) as did the documented VTE risk scores (the percentage of patients with a documented risk score of 5+ grew from 10.4 to 23.8%; see the Supplementary Material Tables S4 and S5 for detailed findings). A further examination showed that changes in risk score were due to increases in prevalence of several risk factors, but mainly these six: age 61 to 74, history of VTE, malignancy, congestive heart failure, abnormal pulmonary function, and sepsis.

**Table 5:** Adjusted VTE rates by CDS stage for total study population and for medical and surgical subgroups

	Total study population—adjusted VTE rate (95% CI)		
CDS stage	Total VTE	In-hospital VTE	Post-discharge VTE
Stage 1	1.11% (1.00–1.23%)	0.55% (0.47–0.64%)	0.56% (0.48–0.65%)
Stage 2	0.92% (0.87–0.97%)	0.33% (0.33–0.36%)	0.59% (0.55–0.63%)
Stage 3	1.13% (1.01–1.24%)	0.41% (0.34–0.48%)	0.72% (0.63–0.82%)
Stage 4	1.07% (0.94–1.19%)	0.27% (0.21–0.33%)	0.80% (0.69–0.90%)
	Medical subgroup—adjusted VTE rate (95% CI)		
CDS stage	Total VTE	In-Hospital VTE	Post-discharge VTE
Stage 1	0.73% (0.60–0.85%)	0.36% (0.25–0.47%)	0.50% (0.39–0.60%)
Stage 2	0.70% (0.65–0.76%)	0.27% (0.23–0.32%)	0.54% (0.49–0.59%)
Stage 3	1.01% (0.87–1.15%)	0.38% (0.27–0.48%)	0.75% (0.62–0.87%)
Stage 4	1.14% (0.98–1.30%)	0.29% (0.20–0.39%)	0.92% (0.77–1.06%)
	Surgical subgroup—adjusted VTE rate (95% CI)		
CDS Stage	Total VTE	In-Hospital VTE	Post-discharge VTE
Stage 1	1.67% (1.45–1.90%)	1.02% (0.84–1.20%)	0.66% (0.51–0.80%)
Stage 2	1.31% (1.21–1.41%)	0.63% (0.56–0.71%)	0.67% (0.60–0.75%)
Stage 3	1.30% (1.11–1.49%)	0.61% (0.48–0.74%)	0.69% (0.55–0.83%)
Stage 4	0.95% (0.76–1.14%)	0.36% (0.24–0.47%)	0.59% (0.44–0.74%)

Abbreviations: CDS, clinical decision support; CI, confidence interval; VTE, venous thromboembolism.

Note: Adjusted VTE rate %.

Finally, patients with smart order set-recommended prophylaxis had a significantly lower adjusted VTE incidence rate in each of the four stages of CDS than patients with orders for insufficient prophylaxis. The differences were largest during stages when risk assessment was voluntary. Differences in adjusted incidence rates of in-hospital and post-discharge VTE between the two prophylaxis subgroups followed a similar pattern. Interestingly, the incidence of post-discharge VTE grew for each subgroup over time ( Table 6 ).

**Table 6:** Adjusted VTE incidence per 10,000 patients by stage of CDS implementation

	Adjusted VTE incidence per 10,000—all VTE		
CDS stage	Smart order set-recommended p	Insufficient prophylaxis	*p-* Value of difference
Stage 1	125	145	<0.001
Stage 2	105	112	<0.001
Stage 3	125	151	<0.001
Stage 4	112	122	0.002
	Adjusted VTE incidence per 10,000—in-hospital VTE		
CDS stage	Smart order set-recommended prophylaxis	Insufficient prophylaxis	*p-* Value of difference
Stage 1	60	78	<0.001
Stage 2	38	43	0.004
Stage 3	45	67	<0.001
Stage 4	26	29	0.403
	Adjusted VTE incidence per 10,000—post-discharge VTE		
CDS stage	Smart order set-recommended prophylaxis	Insufficient prophylaxis	*p-* Value of difference
Stage 1	65	67	<0.001
Stage 2	67	67	0.981
Stage 3	80	83	<0.001
Stage 4	86	89	<0.001

Abbreviations: CDS, clinical decision support; VTE, venous thromboembolism.

## Discussion

This cross-sectional study of CDS for VTE prevention for nearly 225,000 patients is the first to directly examine the difference in impact of mandatory versus voluntary risk assessment on rates of VTE prophylaxis and outcomes, as evidenced by findings from a recent systematic review of experimental and observational studies of the effect of CDS hard stop alerts in health care settings. 31

Study results demonstrate that CDS with mandatory risk assessment produced more improvement in smart order set-recommended prophylaxis and rates of VTE. They also show that the improvements in rates of VTE were due to reductions in VTE that occurred in-hospital; rates of VTE post-discharge actually grew.

When CDS was first introduced, providers documented risk assessment for only 42.4% of patients. With the addition of the hard stop, better integration of risk assessment into the provider workflow, and intensive provider education, the percentage increased in subsequent stages to almost 98% in Stage 4.

Janus and colleagues found that the impact of implementation of CDS with voluntary risk assessment on rates of prophylaxis is highly dependent on documentation of risk assessment; patients with risk assessment had 1.44 times higher odds of receiving risk-appropriate prophylaxis. 22 Our study measured orders for any VTE prophylaxis and showed that patients with a documented risk assessment had significantly higher rates of prophylaxis in CDS with both voluntary and mandatory risk assessment.

The reliability of the provider-documented risk level was poor, regardless of the CDS stage; 44 to 54% of patients had a documented risk level that did not match the risk level calculated using patient risk factors identified from hospital information systems. Of the patients with mismatched scores, the majority had documented risk levels that were lower than the calculated risk levels. Similar findings were reported in several studies of point-scoring systems where a significant percentage of at-risk patients were misclassified as being low risk. 19 20

It is not possible to determine which risk level is more accurate because there are obstacles to calculating risk scores and levels using both methods. The CDS checklist takes time to complete and busy providers may produce incomplete risk assessments with underestimated risk scores. Data from hospital information systems are not complete and cannot provide details about risk factors that can only be observed during face-to-face provider–patient interactions, such as family history of VTE. 20 32 33

To improve the accuracy of CDS risk scores, Borab and colleagues proposed prepopulating the checklist of risk factors based on data in the EHR (hospital information systems) and allowing physicians to add and modify the list. 21 Caprini recommends that patients complete a simplified risk assessment form that providers can verify with the patient when completing the CDS checklist. These approaches could increase both the efficiency and accuracy of risk scoring. 34

The time-series analysis demonstrated that adherence to smart order set-recommended prophylaxis improved with mandatory CDS in Stage 2 and fell back in Stage 3 with a reversion to voluntary CDS, mostly due to delays in orders for prophylaxis. These changes were accompanied by corresponding and inverse changes in the percentage of patients with orders for insufficient prophylaxis.

The reintroduction of mandatory CDS in Stage 4 did not produce a statistically significant increase in smart order set-recommended prophylaxis because increases occurred after implementation of Stage 3 voluntary risk assessment, possibly due to QI interventions such as alerts and feedback to providers about observed problems with insufficient prophylaxis. 35 By Stage 4, the rate of smart order set-recommended prophylaxis recovered from the lapse that occurred with implementation of Stage 3. By the end of the study period, adherence to smart order set-recommended prophylaxis was about 80%.

The mandatory CDS introduced at UMH forced risk assessment for every patient. Studies show that mandatory CDS tools requiring providers to proactively select risk factors increase rates of risk-appropriate prophylaxis and are more effective than voluntary or passive tools that do not require provider action. 36 37 A meta-analysis of CDS for surgical patients showed that mandatory risk assessment was the “vital function” to ensuring statistically significant increases in risk-appropriate prophylaxis. 21

The same meta-analysis found that auto-population of orders for prophylaxis with an opt-out feature produced optimal rates of appropriate prophylaxis. 21 At UMH, CDS was designed to encourage providers to order risk-appropriate prophylaxis or opt out beginning in Stage 2, which contributed to higher rates of smart order set-recommended prophylaxis.

In our study, documentation of risk assessment led to substantial increases in orders for prophylaxis and the documented risk level was underestimated for a significant percentage of patients. These findings raise questions about the potential for inappropriate prophylaxis, particularly in patients with documented low risk, the only risk level where the smart order set does not recommend prophylaxis.

We found that inappropriate (excessive) prophylaxis was small because the percentage of low-risk patients was small, varying from Stages 1 to 4: 13, 23, 14, and 15%. Of these patients, about 1/3 had orders for chemoprophylaxis, 1/3 had orders for sequential compression devices, and 1/3 had no orders for prophylaxis.

A detailed examination of low-risk patients showed that 47 to 91% had a higher risk level based on data from hospital information systems. This suggests that a large portion of low-risk patients with orders for prophylaxis were moderate or high risk. Consequently, measures of excessive prophylaxis may be less meaningful than measures of insufficient prophylaxis for evaluating inappropriate prophylaxis.

Analyses of VTE outcomes showed that rates of adjusted VTE and adjusted odds ratios of VTE in pairwise comparisons were lower in stages with mandatory compared to voluntary risk assessment. Increases in the percentage of patients with smart order set-recommended prophylaxis during stages with mandatory risk assessment contributed to these results.

Our study rigorously followed CHEST guidelines to develop the CDS smart order set recommendations for VTE prophylaxis. The CHEST guidelines did not reflect results from research that proposed raising the Caprini risk score threshold for chemoprophylaxis for different patient subgroups. 25 26 However, we tested the impact of mandatory risk assessment on VTE outcomes for a subgroup of patients with higher Caprini risk scores (scores of 5 + ) and observed that increasing the risk score threshold for chemoprophylaxis did not affect our finding that implementation of mandatory VTE risk assessment with recommendations for prophylaxis is key to reducing VTE outcomes.

Our study results show that the lower rates of adjusted VTE in stages with mandatory risk assessment were driven by lower rates of in-hospital VTE. By contrast, post-discharge VTE rates increased from one stage to the next. The adjusted incidence rate of post-discharge VTE for patients with smart order set-recommended prophylaxis grew similarly.

Although studies of HA-VTE published during the early stages of our CDS implementation showed that a majority of 90-day VTE outcomes occurred after discharge and were attributed to suboptimal inpatient prophylaxis and/or lack of extended post-discharge prophylaxis for some high-risk patients, 2 3 38 the primary focus of our study was on improving inpatient prophylaxis consistent with the CHEST guidelines.

Missed administration of recommended inpatient prophylaxis could have contributed to the increase in post-discharge VTE; however, in-hospital VTE decreased during the study period and QI initiatives, including nurse and patient education about the importance of VTE prophylaxis and EHR triggers designed to fix problems with missed administrations in real-time, began during Stage 2 and were enhanced over time.

Heit et al studied Olmsted County residents admitted to a Mayo Clinic hospital from 2005 to 2010 and found that adjusted hospital-related VTE rates within 90 days did not change significantly despite dramatic improvements in the rate of risk-appropriate prophylaxis during hospitalization. The lack of improvement in VTE rates was due to post-discharge VTE, which accounted for 75% of all VTE. Unfortunately, the relative contribution of surgical and medical patients to VTE rates was not reported. 39

In a study of general medical patients, rates of VTE within 90 days were not appreciably different between hospitals with high rates of pharmacologic prophylaxis (85.8%) and those with much lower rates (55.5%). Eighty-five percent of VTE occurred after discharge. 40 Studies of surgical populations showed a discordance between VTE outcomes and adherence to national VTE prophylaxis standards, 41 42 indicating that improvements in short-term postoperative prophylaxis do not lower VTE rates.

In our study, post-discharge VTE accounted for about 50% of all VTE in Stage 1 and over 70% in Stage 4. If potentially preventable VTE is defined as VTE within 90 days in patients who received insufficient prophylaxis, 13 43 then a significant proportion of post-discharge VTE was probably not preventable. These findings indicate that inpatient-only VTE prevention strategies are no longer sufficient and evaluation of post-discharge risk and extended prophylaxis for vulnerable patients must be considered to further reduce the burden of VTE.

The increase in post-discharge VTE in our study population was driven by the subgroup of medical patients. Studies show that patients with acute medical illnesses are at risk for VTE after discharge, owing to continued risk after discharge, underuse of in-hospital prophylaxis, or decreasing hospital lengths of stay leading to decreasing duration of in-hospital prophylaxis. 44 45 In our study, medical patients had an increase in hospital lengths of stay and patient risk. The increased duration of in-hospital prophylaxis for these patients was not sufficient to counteract their higher risk of VTE from being bedridden for longer periods and experiencing more comorbid illnesses.

Our primary goal was to successfully implement CDS for inpatient prophylaxis by applying uniform, evidence-based guidelines. This goal was achieved and in-hospital VTE decreased significantly. Our next focus will be on identifying patients for extended prophylaxis to reduce VTE post-discharge. Current guidelines do not support routine prescribing of post-hospital prophylaxis for acutely ill medical patients because the benefit of VTE reduction from extended anticoagulation may be offset by the harm of major hemorrhagic complications. 44 However, three clinical trials provided evidence of the efficacy and safety of two agents, betrixaban and rivaroxaban, for preventing VTE and VTE-related death in these patients and two adopted strict eligibility criteria to exclude patients with conditions indicating a higher risk for bleeding. 46 47 48

The North American Thrombosis Forum Anticoagulation Action Initiative proposed that the eligibility criteria from these trials be used at discharge to identify high risk medically ill patients for post-discharge prophylaxis. 49

The increase in risk of medical patients in our study was associated with substantial increases in the prevalence of six Caprini risk factors commonly found in the eligibility criteria for the trials. To implement a program to identify acutely ill medical patients for longer duration post-discharge prophylaxis will require refining the Caprini VTE risk stratification and combining it with systematic scoring of bleeding risk. 44

Boston Medical Center (BMC), using the Caprini RAM to implement mandatory CDS for VTE prevention in general and vascular surgery patients, established a Caprini VTE risk score threshold of 5 for chemoprophylaxis that included routine extended, post-discharge chemoprophylaxis. Instead of using CHEST guidelines, their prophylaxis recommendations were based on evidence in published studies of the incidence of VTE by Caprini risk level in general and vascular surgery patients and of the efficacy of extended, post-discharge prophylaxis in high-risk patients. The BMC mandatory CDS was designed very similarly to ours and investigators found that the CDS contributed to significant improvements in 30-day VTE outcomes. Although the BMC study did not include medical patients or report the incidence in-hospital versus post-discharge VTE and bleeding outcomes, it confirmed that implementation of a mandatory, standard evidence-based protocol for risk assessment and prophylaxis reduces the incidence of VTE. 50

The AHA call to action established a goal of reducing VTE in hospitalized patients by 20% by 2030. It specified steps for standard measurement and tracking of hospital rates of risk assessment, prophylaxis, VTE, and preventable VTE. 10 But, with high rates of post-discharge VTE, decreasing hospital lengths of stay, and increasing comorbidity of hospitalized patients, we recommend AHA adopt additional steps aimed at establishing national guidelines for extended duration, post-discharge prophylaxis.

There are several limitations to this study but also some notable strengths. First, it was conducted for a CDS system implemented at one academic center. Nevertheless, it examined real-world data for almost 225,000 patients over 8 years and is the first to provide direct evidence of the superiority of mandatory versus voluntary risk assessment in increasing smart order set-recommended prophylaxis and reducing VTE outcomes and is supported by an evaluation of a similar mandatory system at BMC. 50 It also makes a valuable contribution to the literature about the persistence of HA-VTE despite high rates of in-hospital VTE prophylaxis. Second, rates of bleeding outcomes were not analyzed to assess the impact of each CDS stage on complications of chemoprophylaxis. Although the CDS allowed physicians to opt-out of smart order set-recommended prophylaxis for patients at high risk for bleeding, this limitation is relevant because of observed problems with the accuracy of risk scoring and recent studies demonstrating that the benefit/harm of chemoprophylaxis for some populations was favorable only in patients with higher Caprini risk score thresholds than were used in our study. 27 Third, post-discharge VTE events were identified based on data from UMH. If patients were diagnosed with post-discharge VTE at an outside facility, the outcome would not be counted unless it was also captured in UMH systems. Although some VTE events after discharge may have been missed, our study showed that reported rates of post-discharge VTE were substantial and represented a large opportunity for improvement. Fourth, the study evaluated orders for VTE prophylaxis, not administration of prophylaxis. Ordering prophylaxis does not ensure its administration, but the multifaceted UMH program for VTE prevention helps align orders with administrations. Fifth, our study could not quantify the specific impact of mandatory risk assessment independent of concomitant QI interventions for increasing risk assessment and risk-appropriate prophylaxis. However, most QI interventions were in effect throughout the study, and we attempted to gauge the influence of others that were added during later stages of CDS implementation on our results.

## Conclusion

Findings from this study of the UMH CDS for VTE prevention demonstrate that mandatory risk assessment is more effective than voluntary risk assessment in improving adherence to smart order set-recommended prophylaxis and rates of VTE, particularly in-hospital VTE. Improvements in the CDS are needed to increase the efficiency and accuracy of risk scoring and to update risk thresholds for chemoprophylaxis based on recent evidence.

Rates of post-discharge VTE contributed substantially to overall rates even with high adherence to risk-appropriate inpatient prophylaxis. These findings indicate that there is an unmet need for national guidelines for extended prophylaxis to prevent VTE post-discharge.
