# Supplementary material for: Impact of Clinical Decision Support with Mandatory versus Voluntary Venous Thromboembolism Risk Assessment in Hospitalized Patients
Source: TH Open. 2024 Sep 12;8(3):e317–28. doi: 10.1055/s-0044-1790519 (PMC11392591; doi:10.1055/s-0044-1790519)
Supplement: Supplementary file 1 — Supplementary Material [file 10-1055-s-0044-1790519-s24040012.pdf]

Supplementary Material

Supplemental Background—UMH CDS and QI Programs for VTE Prevention

Features of the UMH CDS (University of Michigan Health clinical decision support) during the four stages of its evolution are summarized in the following table.[20] Key among them was the use of an opt-out feature to compel the provider to select risk appropriate prophylaxis, beginning in Stage 2. These "smart order sets"[13] were modified for some populations during and after this study to recalibrate the Caprini risk score threshold for chemoprophylaxis based on evidence that a favorable benefit/risk ratio of chemoprophylaxis is achieved with higher risk scores.[26]

Supplementary Table S1 Features of clinical decision support by stage of implementation

CDS feature	Stage of implementation			
	Stage 1—Eclipsys CPOE Voluntary	Stage 2—Eclipsys CPOE Mandatory	Stage 3—EPIC EHR Voluntary	Stage 4—Epic EHR Mandatory
Foundation System with Embedded CDS	Eclipsys CPOE	Eclipsys CPOE	Epic EHR	Epic EHR
Page Links for Risk Assessment	Admission, preoperative	Admission, preoperative	Admission, perioperative	Admission, perioperative
Integration into Workflow (Y/N)	N	Y	Y	Y
Voluntary/Mandatory Use	Voluntary	Mandatory	Voluntary	Mandatory
Risk Factors Entered/Prepopulated	Entered	Entered	Entered (only patient age prepopulated)	Entered (only patient age prepopulated)
Link to VTE Guidelines (Y/N)	N	N	Y	Y
Contraindications to Chemoprophylaxis Captured	Y	Y	Y	Y
Specialty/Population-Specific Order Sets (Y/N)	N	Y (trauma surgery)	Y (trauma surgery, inflammatory bowel disease)	Y (trauma surgery, inflammatory bowel disease, obesity)
Guideline-Recommended Orders Presented (Y/N)	Y	Y	Y	Y
Guideline-Recommended Orders: Opt-In or Opt-Out	Opt-in	Opt-out	Opt-out	Opt-out
Electronic Alert for Inadequate Prophylaxis	N	Y	Y	Y
Information Saved for Analysis (Y/N)	Y	Y	Y	Y

Abbreviations: CPOE, computerized physician order entry; EHR, electronic health record; Y, yes, the feature is present; N, no, the feature is not present.

UMH adopted a multifaceted approach to venous thromboembolism (VTE) prevention. In addition to employing CDS, UMH initiated quality improvement (QI) interventions, including performance feedback to physicians about orders for risk-appropriate prophylaxis, audits of missed administrations of chemoprophylaxis, and CDS alerts to caregivers when orders are for insufficient prophylaxis and when patients refuse chemoprophylaxis, nurse and patient education programs to increase use and documentation of VTE prophylaxis and real time triggers to correct defects in orders or administration of VTE prophylaxis. In the hierarchy of reliability describing different stages of QI efforts, UMH achieved a score of 4 of a possible 5 and a predicted performance of 90% in achieving appropriate prophylaxis.[8] The following table lists the QI interventions during the study period.

 © 2024. The Author(s).

Supplementary Table S2 Quality improvement interventions by stage and date of implementation

CDS Stage	Start and end dates		Quality improvement intervention
	Start	End	
1–4	2008	Ongoing	Provider Education and Roll-Out of CDS for VTE Prevention<br>Provider VTE and VTE prevention education, with oversight by the Executive Committee for Clinical Affairs using a variety of mechanisms (directed communication from service chiefs, newsletters, etc.). Also, training to use order entry to conduct risk assessment and write orders for VTE prophylaxis within the workflow for patient admission.
1	Oct 2008	May 2009	CDS Alerts<br>Risk Assessment Reminder Alert<br>Alert to provider within 24 hours after admission when patient has no documented risk score. Occurs each time a provider uses order entry.
1–4	Jan 2009	Ongoing	VTE Prevention Committee<br>Committee with multidisciplinary representation (e.g., physicians from several specialties, nurses, QI and IT specialists) established to develop standard policies, protocols, and guidelines for VTE prevention and to review UMH performance data and to design and initiate interventions to improve VTE risk assessment, prophylaxis, and outcomes.
1–4	May 2009	Ongoing	Monthly Performance Feedback for VTE Committee<br>Feedback about UMH overall and specialty-specific rates of risk assessment completion/timeliness and prophylaxis and overall rates of in-hospital and 90-day post-discharge VTE.
2–4	Jun 2009	Ongoing	Quarterly Individual Physician Feedback<br>As part of the Ongoing Professional Performance Evaluation (OPPE) program, rates of risk-appropriate VTE prophylaxis (and later, rates of VTE outcomes) were produced for individual physicians in adult medical and surgical specialties.
2	Aug 2009<br>Feb 2011<br>Dec 2009<br>Jun 2010	May 2014<br>May 2014<br>May 2014<br>May 2014	CDS Alerts<br>Postop Risk Assessment Reminder Alert<br>Alert sent to provider when patient operation is completed with request to confirm accuracy of preoperative VTE risk assessment.<br>No Chemoprophylaxis Orders Alert<br>Alert to provider when patient has moderate to high risk level and no chemoprophylaxis orders with reminder to initiate chemoprophylaxis if clinically appropriate.<br>Insufficient Chemoprophylaxis Alert<br>Alert to provider when patient has moderate to high risk level and an order for BID unfractionated heparin to consider changing order to TID unfractionated heparin, if clinically appropriate.<br>Contraindication Reminder Alert<br>Alert to provider within 24 and 48 hours after admission, when patient has moderate to high risk level and documented contraindication to chemoprophylaxis, to reassess for chemoprophylaxis.<br>Patient Refusal Alert<br>Alert sent to nursing to page provider primary service if patient refuses scheduled chemoprophylaxis dose.<br>Hold/Suspend Prophylaxis Alert<br>Alert to provider when patient has a high-risk level and an order to suspend chemoprophylaxis to assess for restarting chemoprophylaxis.
2	Sep 2010	Ongoing	Conduct Routine Root Cause Analysis of VTE Outcomes<br>VTE Prevention Committee member review of in-hospital VTE to identify root causes involving lapses in VTE risk assessment and prophylaxis and to develop and initiate improvement plans.
2	Jan 2012	Ongoing	Nurse and Patient Education<br>Routine nursing education expanded to address occurrence of missed heparin administrations and lack of documentation about use of mechanical prophylaxis. Included simulations and written tests and information to help nurses reduce patient refusal of VTE prophylaxis by informing patients about the importance of prophylaxis. Patient education supported by UMH video accessible on-demand from the hospital room television (Patient Education Channel).

 © 2024. The Author(s).

Supplementary Table S2 (*Continued*)

CDS Stage	Start and end dates		Quality improvement intervention
	Start	End	
3	Jun 2014<br>Jun 2014<br>Jun 2014	Aug 2015<br>Aug 2015<br>Ongoing	CDS Alerts<br>Risk Assessment Reminder Alert<br>Alert sent to provider 24 hours after admission for patients without VTE risk assessment, to conduct risk assessment. Continues until assessment is completed.<br>VTE Prophylaxis Order Alert<br>Alert sent to provider when patients with moderate to high risk level have no order for chemoprophylaxis or documented contraindication with reminder to complete VTE order set. Alert appears on order entry.<br>Sequential Compression Device (SCD) Reminder Alert<br>Alert to nurses if patient has active order for mechanical prophylaxis but no nursing documentation of application within 6 hours. Continues every 6 hours.
3	Jun 2014	Ongoing	Nurse Education<br>Nurse education program to increase use and documentation of SCDs.
4	Sep 2015	Ongoing	Feedback Report of Potentially Preventable VTE<br>Feedback report created for VTE Prevention Committee to display trends in in-hospital VTE that were potentially preventable due to lapses in orders for VTE prophylaxis or missed administration of chemoprophylaxis.
4	Nov 2015	Ongoing	Alerts for Real-Time Intervention<br>Alerts with patient lists sent to providers, pharmacists, and nurses to correct lapses in orders or administration discovered using active surveillance. Alerts to providers to reassess patients with moderate to high-risk level and documented contraindication to chemoprophylaxis, to reassess for chemoprophylaxis. Alerts to nurses when patient refuses scheduled chemoprophylaxis dose or when the patient has an active order for mechanical prophylaxis with no documentation of application.

Abbreviations: BID, twice daily; CDS, clinical decision support; TID, thrice daily; VTE, venous thromboembolism.

 © 2024. The Author(s).

Demographic Characteristics of Study Population

Supplementary Table S3 Demographic characteristics of study population overall and by stage

	All ($n = 223{,}405$)	Stage 1 ($n = 29{,}520$)	Stage 2 ($n = 134{,}013$)	Stage 3 ($n = 32{,}239$)	Stage 4 ($n = 27{,}633$)
Sex					
Female	119,369 (53.4%)	15,282 (51.8%)	74,143 (55.3%)	16,129 (50.0%)	13,815 (50.0%)
Male	104,035 (46.6%)	14,237 (48.2%)	59,870 (44.7%)	16,110 (50.0%)	13,818 (50.0%)
Unknown	1 (0.0%)	1 (0.0%)	0 (0.0%)	0 (0.0%)	0 (0.0%)
Age					
Mean [SD]	53.7 [18.6]	53.9 [18.4]	52.7 [18.9]	55.6 [17.8]	55.9 [17.8]
18–40	59,454 (26.6%)	7,287 (24.7%)	39,427 (29.4%)	6,847 (21.2%)	5,893 (21.3%)
41–60	75,597 (33.9%)	10,981 (37.2%)	44,118 (32.9%)	11,293 (35.0%)	9,364 (33.9%)
61–74	57,825 (26.0%)	7,073 (24.0%)	32,727 (24.4%)	9,732 (30.2%)	8,451 (30.6%)
≥75	30,212 (13.5%)	4,179 (14.2%)	17,741 (13.2%)	4,367 (13.6%)	3,925 (14.2%)
Subgroup					
Medical	139,667 (62.5%)	17,459 (59.1%)	85,659 (63.9%)	19,149 (59.4%)	17,400 (63.0%)
Surgical	83,738 (37.5%)	12,061 (40.9%)	48,354 (36.1%)	13,090 (40.6%)	10,233 (37.0%)

Abbreviation: SD, standard deviation.
Note: Categorical data are shown as count (%), and continuous data are shown as mean (SD).

Results from Examination of Increase in Post-discharge VTE

Supplementary Table S4 Mean hospital length of stay for total study population and for medical and surgical subgroups

	Mean length of hospital stay in days		
CDS stage	Total study population	Medical subgroup	Surgical subgroup
Stage 1	6.0	5.3	7.1
Stage 2	5.4	4.8	6.6
Stage 3	5.7	5.5	6.0
Stage 4	5.8	6.4	4.9

Abbreviations: CDS, clinical decision support.
Note: Data shown as mean.

 © 2024. The Author(s).

Supplementary Table S5 Distribution of Caprini risk score for total study population and for medical and surgical subgroups

	**Total study population—Caprini risk score**			
CDS stage	0	1–2	3–4	5+
Stage 1 (12,513)	0.0% (0)	11.6% (3,417)	14.4% (4,244)	16.5% (4,852)
Stage 2 (122,653)	0.0% (0)	34.8% (46,658)	29.5% (39,508)	27.3% (36,487)
Stage 3 (29,513)	4.5% (1,438)	20.0% (6,435)	34.5% (11,129)	32.6% (10,511)
Stage 4 (27,002)	4.9% (1,347)	21.6% (5,976)	36.9% (10,191)	34.3% (9,488)
	Medical subgroup—Caprini risk score			
CDS stage	0	1–2	3–4	5+
Stage 1 (7,315)	0.0% (0)	16.0% (2,796)	15.5% (2,708)	10.4% (1,811)
Stage 2 (77,002)	0.0% (0)	45.1% (38,602)	29.7% (25,448)	15.1% (12,952)
Stage 3 (17,834)	6.4% (1,223)	25.9% (4,959)	38.3% (7,332)	22.6% (4,320)
Stage 4 (16,902)	6.5% (1,134)	26.5% (4,608)	40.3% (7,015)	23.8% (4,145)
	Surgical subgroup—Caprini risk score			
CDS stage	0	1–2	3–4	5+
Stage 1 (5,198)	0.0% (0)	5.2% (621)	12.7% (1,536)	25.3% (3,041)
Stage 2 (45,651)	0.0% (0)	16.7% (8,056)	29.1% (14,060)	48.7% (23,535)
Stage 3 (11,679)	1.6% (215)	11.3% (1,476)	29.0% (3,797)	47.4% (6,191)
Stage 4 (10,100)	2.1% (213)	13.4% (1,368)	31.0% (3,176)	52.2% (5,343)

Abbreviation: CDS, clinical decision support.
Note: Categorical data are shown as % (count).

 © 2024. The Author(s).
